# Stress during Home Confinement Is Associated with Eating Misalignment among Adults during COVID-19 Lockdown

**DOI:** 10.3390/nu15184018

**Published:** 2023-09-16

**Authors:** Abeer M. Aljaadi, Rinal J. Bogis, Nouf A. Alruhili, Saja O. Alharbi, Essra A. Noorwali

**Affiliations:** Clinical Nutrition Department, Faculty of Applied Medical Sciences, Umm Al-Qura University, Makkah 24381, Saudi Arabiaeanoorwali@uqu.edu.sa (E.A.N.)

**Keywords:** COVID-19, Saudi Arabia, dietary habits, eating misalignment, weight, home confinement, curfew, lockdown, stress, sleep quality

## Abstract

Background: The COVID-19 pandemic forced Saudi Arabia to implement several measures including mandatory home confinement, banning entry to many cities, and suspending religious activities. Studies have reported inconsistent findings of the effect of home confinement on lifestyle factors. This study aims to assess the psychological impact of COVID-19 during home confinement and explore its association with dietary habits and weight change. Methods: A cross-sectional study was conducted among Saudi adults using an online survey between May and June 2020. Data on dietary habits, sleep quality, and stress were collected. Results: A total of *n* = 503 participants responded. Of 254 analyzed, 87% were females, 49% were overweight/obese (body mass index (BMI) ≥ 25 kg/m^2^), and 79% were under lockdown for >40 days. In multiple linear regression, higher stress scores during confinement were associated with higher stress scores before confinement and poorer sleep quality. In multiple logistic regression, those who did not eat at the same time had higher stress scores compared to those who always ate at the same time, whereas consuming ≥three meals was associated with lower stress scores than consuming one–two meals. The odds of gaining weight during confinement were higher among married adults, those with lower sleep quality, and consuming ≥three meals. Conclusions: Stress during home confinement was associated with eating misalignment and the number of meals consumed. Although this study was limited by its cross-sectional design and reliance on self-reported data, it provides valuable insights into the dietary habits and weight-gain associated factors that need to be further explored and addressed in any future restrictions for improved well-being.

## 1. Introduction

On 11 March 2020, the World Health Organization designated the coronavirus disease of 2019 (COVID-19) emergency to be a global pandemic [1]. Many governments of countries that were extensively affected implemented policies that restricted social communication to limit COVID-19 spread, including Saudi Arabia [2]. Saudi Arabia implemented a range of measures including a curfew, mandatory home confinement, banning entry to many cities, and suspending religious activities starting 9 March 2020 [2]. Despite the success in controlling COVID-19 spread, the mandated restrictions adversely affected lifestyle behaviors, such as poor sleep quality, poor dietary habits, socioemotional disorders, weight gain, increased screen time, and lack of physical activity in several countries [3,4,5,6]. Therefore, studying the impact of home confinement on lifestyle behaviors is essential to prevent adverse changes in future pandemics.

Several studies reported mental health problems during the COVID-19 pandemic [7,8]. The main mental health stressors during the quarantine included the duration of the quarantine, fears of infection, frustration and boredom, inadequate supplies, and inadequate information [9]. Stress can lead to health problems including an increase in blood pressure, blood glucose, and body weight. Studies have shown that persistent stress weakens the immune system via increasing glucocorticoids and pro-inflammatory biomarkers, such as interleukin-6 [10]. Higher cortisol levels are associated with a greater consumption of foods, and thus weight gain [11]. Consequently, assessing the impact of stress on dietary behaviors during home confinement needs to be addressed.

Several studies have demonstrated heterogeneity in eating behaviors and weight change in response to stress; some people eat more when stressed while others eat less. Moreover, different factors during COVID-19 lockdowns could influence the relationship between stress and dietary habits or weight gain, including alterations in physical activity, screen time, sleep quality, and employment status. Higher stress has been generally associated with increased caloric intake, snacking, and consumption of high fat/sugar foods [12]. However, the consumption of homemade meals and breakfast throughout the pandemic increased, and the percentage of those eating mostly fast food and skipping meals decreased [13,14,15,16,17]. Whether dietary changes during the COVID-19 pandemic were stress-related is not clearly understood. Individuals who reported restricted eating, skipping meals, or overeating during the pandemic reported having higher stress levels, after adjusting for sex, age, race, ethnicity, employment, marital status, the region in the US, and BMI [18]; sleep quality during the outbreak and stress level before confinement were not accounted for, which could influence the relationship [16,19].

Increased sitting and screen time, and exposure to stress related to lockdown were correlated with weight increase [17,20], but these studies did not control for potential confounders. Perceived stress scores during the COVID-19 outbreak were not associated with weight increase among women in Riyadh, after adjusting for age, education, sleep quality, physical activity, and baseline stress [16]. A study on U.S. adults reported that weight gain did not correlate with perceived stress scores in bivariate analysis [21]. Some studies have reported better sleep, increased physical activity, and improved diet quality during the pandemic [4,13,14], while others reported worsened lifestyle changes. According to a study conducted on 838 U.S. adults during the last week of April 2020, mean stress scores, as measured by the perceived stress scale, varied by ethnic groups, were higher among people who had part-time jobs, were single, and were younger than 35 years [18]. Inconsistent results between studies could be due to the lack of controlling for such confounding factors, and more research is needed to explore the relationship between stress and changes in dietary habits and weight status. Therefore, the present study aimed to explore the psychological impact of COVID-19 home confinement on dietary habits and weight change taking into consideration changes in sleep quality and physical activity.

## 2. Materials and Methods

### 2.1. Study Design

This was a cross-sectional study conducted during the COVID-19 lockdown in Saudi Arabia. Restrictions during COVID-19 were updated almost weekly, with the months of mid-March to May 2020 involving a 24-h curfew and forcing people into home confinement. During home confinement, individuals were not allowed to leave their houses except with an electronic permit [2]. Permit types differed, but all were obtained through a mobile app (Tawakkalna) [22]. Healthcare workers and some employees had exceptions during these lockdowns; however, the majority of the population was under home confinement. Social distancing measures included keeping a distance of at least 1.5 m between people, prohibiting all public gatherings (including religious activities), limiting attendance at private events like weddings and funerals to five people, and forbidding meetings with more than one person who is not a member of one’s immediate family. As part of the lockdown procedures, eating establishments, a number of retail stores, and access to outdoor parks were all prohibited.

A 118-item online survey was administered using Google Form^®^ in Arabic and English languages, which included information on sociodemographic, home confinement, health, sleep quality, stress, physical activity, and eating habits. Several questions were presented in a distinguishing formula with questions that focused on change that occurred “during” compared to “before” the confinement period. Responses after June 2020 were excluded, as several restrictions were lifted on 21 June. Only adults (>18 years) who were living in Saudi Arabia at the time of the survey were included. Pregnant and lactating women were excluded because of their altered stress level, sleep quality, and dietary habits [23,24,25].

### 2.2. Dietary Habits and Physical Activity

A few questions on dietary habits were administered, such as the usual number of main meals consumed, usual number of snacks consumed, frequency of eating at restaurants or take-away or delivered food, frequency of having meals at the same time, and weight change during confinement compared to before confinement. Physical activity was assessed using three questions from the short form of the International Physical Activity Questionnaire [26]. A question on screen time was included by asking about the duration spent in front of screens (phone, computer, TV, etc.) before and during confinement. Body weight and height were self-reported and Body Mass Index (BMI) was calculated as weight (kg)/height (m)^2^. BMI was classified into 3 categories: underweight (<18.5 kg/m^2^), healthy weight (18.5–24.9 kg/m^2^), and overweight/obese (≥25 kg/m^2^) [27].

### 2.3. Sleep Quality and Stress Assessment

Sleep quality was assessed using the Pittsburgh Sleep Quality Index (PSQI). Each element was given a score between 0 and 3, with a total score ranging from 0 to 21. Higher scores indicate disturbed sleep. Perceived sleep quality was assessed using the Pittsburgh Sleep Quality Index (PSQI). Each element was given a score between 0 and 3, with a total score ranging from 0 to 21. Higher scores indicate disturbed sleep [28]. Questions on stress were adopted from the depression subscale in the Calgary Symptoms of Stress Inventory (C-SOSI). The C-SOSI includes 56-item scale and 8 domains (sub-scales). One of the sub-scales, relating to the variety of emotions that accompany depression, was used as an indicator of affective stress responding to home confinement [29]. Questions on the subscale were formatted to “before” and “during” confinement. Response options were as follows: 0 = never, 1 = almost never, 2 = sometimes, 3 = most of the time, 4 = almost always. The total sum of the subscale score was used with higher scores indicating higher stress levels.

### 2.4. Statistical Analyses

Descriptive statistics were used to define means SD, medians [25th, 75th percentiles], or proportions. To assess differences in continuous variables before and during the confinement period, paired sample *t*-tests or Wilcoxon test were used as appropriate. McNemar’s test was used to compare categorical variables before and during confinement. Multiple linear regression was used to determine factors associated with stress during confinement. Logistic regression was used to assess the association between dietary habits and weight change (categorical outcomes) with stress scores and other factors. Models were adjusted for potential confounding variables; variables were included in the model if they had a bivariate correlation of *p* ≤ 0.2 with the outcome. The number of events in the outcome was taken into consideration to limit the number of independent variables included in the logistic regression models [30]. All dietary habit models were adjusted for the dietary habit before confinement and fasting during Ramadan. During Ramadan, Muslims abstain from food and drink between dawn and dusk, with the exception of children under puberty, pregnant and lactating women, the sick and debilitated, and those who are traveling. All data were analyzed using Stata software version SE/14.2 for Mac (Stata Corp., College Station, TX: StataCorp LP) and the statistical significance was set to *p* < 0.05.

## 3. Results

Overall, *n* = 503 participants responded to the survey from 16 May to 28 June 2020. A total of 119 participants (*n* = 119) were excluded as they were not living in Saudi Arabia at the time and we wanted to minimize variability that could be attributed to differences in countries’ restrictions. Participants <19 years old (*n* = 5), pregnant women (*n* = 8), breastfeeding women (*n* = 19), and those who were not under home confinement (*n* = 37) were excluded. After removing duplicates in Stata (*n* = 61), the final sample for analysis was *n* = 254 and their responses were received between 16 May and 12 June 2020.

Participants’ characteristics are shown in Table 1. Mean ± SD age was 31.7 ± 8.8 years. The majority of the participants were females (87%), had a bachelor’s degree or higher (91%), and almost half were married (48%). Moreover, most respondents were Saudis (88%), over half were from the Western region (67%), and 45% had children. The mean ± SD BMI was 26.2 ± 6.5 kg/m^2^, and 49% of the participants were classified as overweight or obese. About 60% (*n* = 153) of the participants reported a change in their weight during confinement; 30.7% reported losing weight, while 29.5% reported gaining weight.

### 3.1. Changes in Stress and Lifestyle Factors during Home Confinement

**Figure 1:**
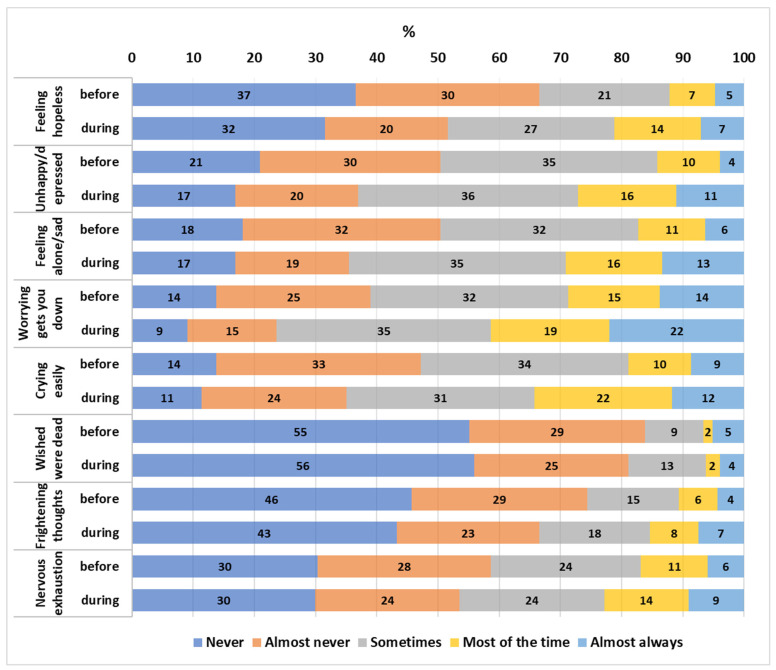
Percentages of stress responses before and during home confinement. All components were statistically significant (*p* < 0.001) before and during confinement, except for “wished were dead”), using Wilcoxon signed-rank test.

Stress components before and during home confinement are presented in Figure 1. Comparisons of stress scores, dietary habits, and other lifestyle factors before and during home confinement are shown in Table 2. Mean ± SD stress scores (depression subscale) during home confinement were higher than before home confinement (12.8 ± 7.5 vs. 10.7 ± 7.3, *p* < 0.0001). Similarly, the mean ± SD PSQI scores were higher during home confinement compared to before, indicating poorer sleep quality (7.3 ± 3.5 vs. 6.3 ± 3.3, *p* < 0.0001). For physical activity, the median number of days spent in walking for at least 10 min was reduced from 2 days before confinement to 1 day per week. However, no significant differences were observed in days spent engaging in moderate or vigorous activities before and during home confinement. Screen time was significantly different during home confinement compared to before; 58% reported spending more than 6 h on their phones, computers, TV, etc. during home confinement compared to 24% before confinement (*p* < 0.001).

Some dietary habits have significantly changed during home confinement compared to before home confinement (Table 2). The proportions of adults consuming ≥three meals compared to one–two main meals decreased during home confinement, whereas consuming ≥two snacks per day increased during home confinement. Moreover, participants were less likely to consume outside food (restaurants, take-away, or delivered food) or eat at the same time during home confinement compared to before. There was no significant change in supplement use during home confinement compared to before confinement.

### 3.2. Predictors of Stress Scores during Confinement

Factors associated with total stress scores during confinement are shown in Table 3. Bivariate analysis showed that higher stress scores during confinement were associated with higher stress scores before confinement, poorer sleep quality, and weight change. However, lower stress scores during confinement were reported by those who spent more days walking and those who have children compared to those who do not have children. In multiple linear regression, only variables that have a *p*-value < 0.2 in the bivariate analyses were included in the model. Sleep quality during confinement, days spent in walking during confinement, and stress scores before confinement remained significantly associated with stress scores during home confinement.

### 3.3. Stress Scores and Eating Habits during Confinement

The association between stress scores during home confinement and eating habits is presented in Table 4. Higher stress scores during home confinement were associated with never/almost never eating food at the same time during confinement, as well as (sometimes) compared to eating food at the same time always or most of the time, adjusting for eating time variable before confinement. Stress scores during confinement remained significantly associated with eating at the same time after adjusting for smoking, sleep quality during confinement, currently fasting during Ramadan, and eating at the same time variable before confinement. Those who never eat at the same time during confinement were more likely to be smokers [OR (95% CI): 5.16 (1.66, 16.00)] and had poorer sleep quality [OR (95% CI): 1.16 (1.02, 1.33)] compared to those who always eat at the same time.

Participants who reported consuming ≥three meals during confinement had lower stress scores during confinement than those who reported consuming one–two meals, adjusting for the number of meals before confinement. This relationship remained significant after adjusting for age, smoking, sleep quality during confinement, currently fasting during Ramadan, and the number of meals before confinement. Consuming outside food (take away, dine out, or delivery) ≥three times per week during confinement was associated with higher stress scores compared to those who reported never consume outside food or less than once a week. This, however, was not significant when adjusting for sex, BMI, marital status, sleep quality during confinement, currently fasting during Ramadan, and smoking.

Gaining or losing weight during confinement was not associated with stress scores during confinement in the bivariate analyses. The odds of gaining weight during confinement was associated with fewer days spent walking for at least 10 min, being married, poorer sleep quality, and consuming ≥three meals compared to one–two meals during confinement (Table 5). Higher odds of losing weight were associated with more days spent doing moderate activity, whereas losing weight was less likely with consuming ≥three meals compared to one–two meals during confinement.

## 4. Discussion

Restrictions imposed to control the COVID-19 pandemic have been reported as negatively affecting lifestyle habits in many countries. These restrictions, however, differed between countries and researchers are still exploring the different impacts to tackle adverse effects on the individual’s well-being in future lockdowns. In this sample of Saudi adults, the majority had been under home confinement for >40 days and reported poorer sleep quality, higher stress scores, and altered dietary habits compared with before confinement. During home confinement, more people consumed only one–two meals, never consumed outside food, and consumed ≥two snacks per day. Higher stress scores during home confinement were associated with never consuming food at the same time, whereas lower stress scores were associated with consuming ≥three meals compared to one–two meals. Weight gain was more likely among those who were married and had poorer sleep quality, but less likely with more days spent in walking.

Approximately 68% of study participants aged 35 years or younger and the majority of our sample were from the Western region that faced more strict regulations, including a 24-h curfew, compared to the rest of the country [2,31], making it an extreme case. However, stress scores during home confinement did not differ between individuals living in the Western region compared to the remainder who did not live in the Western region. The “other regions” category mainly represented participants from the Southern region, which showed higher stress scores during confinement compared to other regions. This relationship did not hold when adjusting for stress scores before confinement, indicating that the stress level before confinement drove the relationship.

We found higher stress scores during home confinement than before confinement, which is in line with previous reports in the Saudi population and other countries [6,13,16,18,19]. High stress scores before confinement predicted high stress scores during confinement, an observation that is expected but not previously reported in the context of COVID-19. As expected, stress during confinement was higher with poorer sleep quality, independent of stress level before confinement. Similar findings were reported during COVID-19 lockdown [19,32,33,34]. Sleep and stress can be bidirectional, with stressors affecting sleep quality and vice versa. Interestingly, those who had children reported lower stress scores during confinement than those who did not have children in bivariate analyses. A study of 4920 healthcare providers in Saudi Arabia during the pandemic reported lower anxiety levels among healthcare providers who have children, but not when adjusting for potential confounders [35]. A study on 838 U.S. adults during the last week of April 2020 reported that mean stress scores, as assessed using the Perceived Stress Scale, differed by ethnic group and were higher among those with part-time jobs, those who were unmarried, and those who were under 35.

Food is a major factor in determining health, and the stress caused by the pandemic influenced how people eat. Several studies have reported changes in dietary habits, but very few have used measures of stress to explore its effect on diets. In our study, dietary habits have changed during the COVID-19 pandemic, including more snacking, variation in the time of eating, fewer main meals, and reduction in consuming foods from outside the home. Some of these changes were associated with higher stress scores during confinement. The frequency of eating food from outside the home was reduced during confinement, similar to other studies in Saudi Arabia [36], which was to be expected given the uncertainty surrounding the safety of outside food in the pandemic and whether the virus could be transmitted through food. Interestingly, those who consumed outside food ≥3 times per week during the confinement tended to have higher stress scores, adjusting for consuming outside food before the pandemic. This relationship was not significant when adjusting for sex, BMI, marital status, sleep quality during confinement, currently fasting during Ramadan, and smoking, but worth further investigation.

In addition to the variation in the time of eating during confinement, higher stress scores during home confinement seem to disturb the day-to-day timing of consuming food. Those who reported never consuming the meals at the same time (eating misalignment) had higher stress scores even after adjusting for smoking, sleep quality, fasting during Ramadan, and dietary habits before home confinement. Stress generally has been reported to affect the quantity of food consumed and food choices [12,37,38], but its impact on eating misalignment is not well-established. The consumption of food at times that contradict our circadian system may have implications for weight status and metabolic health [39,40], and disrupt the synchronization of peripheral clock tissues, such as the liver, leading to metabolic dysfunction [41]. Studies have suggested that shift workers, who often experience circadian misalignment due to irregular sleep–wake and eating patterns, are at an increased risk of developing metabolic syndrome and cardiovascular disease [42]. We believe that no other studies have explored the relationship between stress and consuming meals at set times during the pandemic and this need to be confirmed in other populations, taking into consideration multiple factors [43].

During home confinement, fewer people consumed ≥three meals but more consumed ≥two snacks per day, which could be attributed to skipping meals, as previously reported [6]. However, conflicting findings have been reported on skipping meals. A study in the United Arab Emirates reported lower rates of skipping meals during lockdown and that the lack of time was the main reason for skipping meals before lockdown [13]. A lack of appetite may be the main contributor to skipping meals during the pandemic [6,13]. After adjusting for confounders, mean stress scores remained lower among those who consumed ≥three meals than one–two meals. Similarly, stress in US adults was associated with reducing meals more during the pandemic compared to before [18]. Increased control of over-eating during the lockdown was negatively associated with anxious feelings, but not with depressed mood in Italian adults; questions on anxiety and depression were extrapolated from the Hamilton Anxiety Rating Scale and the Hamilton Depression Scale [44]. Nearly twice the increase in reported anxiety among adults from several countries were observed in association with a perceived change toward unhealthy eating in bivariate analyses [17].

A study on *n* = 1047 adults from several countries reported that unhealthy eating habits and poor sleep were linked to low mental wellness, life dissatisfaction, and high levels of depressive symptoms using multiple questionnaires [34]; it is important to note that only bivariate analyses were conducted in this study without adjustments for any confounders. Binge-drinking alcohol, a negative dietary behavior, was higher in Indian adults (*n* = 354) who were more depressed, as assessed using the Depression Anxiety Stress Scale, during the 2020 COVID-19 lockdown [45]; this study also did not adjust for confounders and used only binary logistic regression. Adults from the US who reported changes in their eating practices (either more or less than before the pandemic) and those reporting that their diet had deteriorated had significantly higher stress scores after controlling for demographic characteristics and BMI [18].

The quality of the diet could be also affected by the stress of the COVID-19 lockdown, but our study focused on dietary habits rather than dietary content. A study on Ecuadorian adults assessing dietary quality during COVID-19 reported higher levels of stress (measured by perceived stress score) with lower dietary quality (measured by the global diet index) [46]. An Italian study concluded that 52.9% were eating more during the lockdown and that there had been an increase in comfort food consumption, including chocolate, ice cream, desserts, and salty snacks [47]. However, not all studies reported low dietary quality during COVID-19 restrictions. A study on 10 Arab countries during the pandemic reported an increase in fruit consumption, unprocessed meats, and lower sugary drinks’ consumption, reflecting positive changes [48]. A similar study in Italy reported a reduction in processed meat and a higher consumption of vegetables, but increased sweets’ consumption [49]. A study conducted in early 2021 on adults in India also reported positive changes in eating habits, such as eating more nutrient-dense food and eating more home-cooked meals, but there was an increase in binge eating, snacking, and larger portion sizes at meals [15]. In US adults, similar findings were reported in terms of a decreased intake of fast food, an increased intake of fruit, and an increased intake of sweets [17]. Dietary factors may help to reduce stress via improving the body’s ability to regulate stress hormones. Overweight and obese women who followed dietary guidelines for 8 weeks had significantly lower levels of stress than those following the traditional American diet; researchers attributed these findings to the combination of higher vegetable intake and a lower sodium intake [50]. Additionally, increasing dietary carbohydrate intake as part of a healthy whole food diet intervention in these women dampened the stress-related changes in salivary cortisol and cortisol responsiveness [51]. Authors suggest that this may be due to the fact that carbohydrate intake helps to stabilize blood sugar levels, which can reduce the body’s need to produce stress hormones.

The Saudi population has been witnessing an increase in poor diets, stress, and obesity over the past few decades, which are associated with adverse health outcomes [52,53]. As a result of the COVID-19 pandemic, it has been proposed that stress and poor diet were exacerbated, creating additional barriers to healthy behaviors. The combined rates of overweight (25%) and obesity (24%) were slightly lower than the national estimates in 2019, when overweight and obesity were 38% and 20%, respectively [53]. Stress is proposed to contribute to overweight and obesity through cognitive processes, behavioral effects, physiological changes in the hypothalamic–pituitary–adrenal axis, and promotion of the production of biochemical hormones and peptides such as leptin, ghrelin, and neuropeptide Y [37]; behavioral effects include overeating, reduced sleep quality, and increased physical inactivity.

Restrictions during COVID-19 in Saudi Arabia were at their highest during the Ramadan and Eid-ul-Fitr holidays, which coincided with our study. This could have affected eating and sleeping habits; however, we controlled for fasting during Ramadan in our dietary models. None of the other studies considered this in their models. Ramadan is a spiritual month in the Arabic lunar calendar practiced by Muslims worldwide and has important social aspects. In 2020, Ramadan was between 23 April to 23 May 2020, and our data collection started on 16 May. In the last ten days of Ramadan, corresponding to 14 May 2020, people were allowed to move freely from 9 A.M. to 5 P.M., in all cities and regions, except for Makkah city where curfew continued [54]. A 24-h curfew was imposed for the first five days of the Eid Al-Fitr holiday (23 May to 27 May 2020) in all cities [55,56]. Intermittent fasting has been shown to affect the circadian system and affect sleep, although subsequent research that took into consideration lifestyle choices and sleep/wake cycles found no differences in biological clock markers, daytime tiredness, or sleep characteristics [57]. The vast cultural and geographic diversity across groups makes it difficult to draw broad conclusions about changes in body weight and diet composition during Ramadan. However, numerous studies show that people do not tend to reduce the number of calories they consume daily during the fasting period [58]. Fasting during Ramadan has been associated with less anxiety in Iranians during the COVID-19 wave in 2021 [59].

Approximately 30% of study participants reported weight gain, which is comparable to the 30.3% in the Middle East and North Africa region [6], and 27.5% worldwide [17]. Estimates in weight gain due to COVID-19-related lockdown varied between populations. For example, weight gain during the lockdown period was 18% in Saudi women [16], 19.5–48.6% in Italy [47,60], 31% in the United Arab Emirates [13], and 22% in US adults [21]. The odds of gaining weight during home confinement were higher among married participants, those with poorer sleep quality, and those with fewer days of walking. Sleep duration has been associated with BMI variation [61]. A study on 173 adults from the US reported that weight gain was associated with shorter sleep duration and less physical activity, but reported null finding with the Perceived Stress Scale [21]. We did not observe sex differences in weight change and most other parameters, which could be because over 70% of the sample were females. Adults over the age of 35 years and males appear to be more likely to gain weight during lockdown [13,48]. However, a study reviewing hospital records from three different regions in Saudi Arabia before and after 2020 reported that females and younger adults have higher odds of gaining ≥5% of their pre-2020 weight; pregnant women do not appear to be excluded, which might have affected these results [62]. Similarly, a study on Danish adults reported that women were more likely to gain weight during the pandemic [14].

Although higher odds of gaining weight were observed with more snacking and a higher number of meals consumed, this was not significant (*p* = 0.06 and 0.07). Young adults who reported overeating to cope with the pandemic gained more weight from baseline to follow-up; however, unhealthy food intake to cope with the pandemic was not associated with weight change [63]. The magnitude of weight gain seems to be a factor, as US adults who gained 5–10 pounds reported more snacking after dinner and eating more in response to stress during the pandemic, but not the other groups of weight change [21]. The odds of losing weight were lower with consuming ≥three meals compared to one–two meals, and higher with increasing days spent in moderate activity. Neither stress scores during confinement nor fasting during Ramadan were associated with odds of weight increase or weight decrease in our sample.

Higher stress scores were associated with higher odds of weight change in our study, but not after adjusting for age, region, sleep quality, physical activity during confinement, and stress score before confinement. A prospective study in Saudi women (*n* = 297) reported no association between stress scores, assessed using the Perceived Stress Scale, and weight change as an outcome in the adjusted models, although baseline stress might be associated with weight gain [16]. A study on adults reported that anxiety scores were higher among those who reported weight change during the lockdown compared to those who stayed the same [17]. Many factors could cause weight gain and loss and conflicting findings between studies could be attributed to not controlling for these factors that could be driving the relationship, and potentially the different tools used to assess stress, anxiety, or depression. A study on *n* = 521 adolescents and young adults in Peru reported that lockdown-related changes in sleep duration, screen exposure time, and sedentarism explained only 6% of the variation in BMI [61]. The weight before confinement was not available in our study and can be a confounding factor in predicting weight change [63]. Other considerations in drawing conclusions on mental health relationships with dietary patterns or weight change are the differences in COVID-19 restrictions as well as timing between nations.

Our study has some strengths. We targeted adults under extremely restrictive conditions during the Spring of 2020. Standardized questionnaires were used to assess sleep, stress, and physical activity, which allowed comparisons with other studies. The questionnaires were administered in English and Arabic to facilitate reachability. Data were collected on before and during home confinement, which provided comparisons and allowed for statistical adjustments of the factors before confinement.

There are some limitations that need to be acknowledged. The cross-sectional nature of the study limits the ability to establish causal relationships between the study’s variables. The reliance on self-reported data may have resulted in some recall, social desirability, and/or recency biases as recent behaviors and emotions would be easier to recall than those that took place in the past. In addition, the sample was collected using an online platform that needed an internet connection and a certain level of literacy to answer the survey, limiting the generalizability of the findings to all adults in Saudi Arabia. For example, people who are less literate or who do not have access to the internet may have been less likely to participate in the study. This could have biased the results towards people who are more educated and have more access to resources. However, this would have a minimal impact given that 98% of individuals in Saudi Arabia had access to the internet in 2020 [64]. Most respondents completed the survey during Ramadan, when all physically capable Muslims are required to fast. Even though it was made explicit in the questionnaire that it was only about the lockdown, it is possible that this had an impact on how participants responded; fasting during Ramadan was adjusted for in the dietary habit models. Moreover, the length of the questionnaire may have introduced a bias towards participants who were more health conscious, as individuals who are highly attentive to their well-being or research-oriented might be more likely to participate and complete the lengthy questionnaire. This self-selection bias could influence the study’s results through overrepresenting individuals who are already more conscious of their health, potentially affecting the generalizability of the findings to the broader population. Details on food items consumed were not available, which limited our ability to adjust for food quantity and energy intake in the weight change models. Specifics about the diet consumed could provide a closer look at the participants’ diet quality and patterns that are factors with a bidirectional relationship with mental health.

## 5. Conclusions

This study explored the lifestyle changes and psychological impact of COVID-19, and the associations between stress, dietary habits, and weight change during the COVID-19 lockdown in Saudi Arabia. Our findings illustrate a significant impact of home confinement on mental health and on unhealthy lifestyle during the COVID-19 pandemic, as opposed to prior confinement. The stress level during home confinement was linked to changes in the timing and number of meals but not weight change. Studies need to consider potential social determinants and confounding factors when studying the impact of lockdowns on the individual’s behavioral and physical changes before drawing conclusions on bivariate analyses. These multifaceted negative impacts emphasize how crucial it is for stakeholders and policymakers to think about creating and putting into practice interdisciplinary solutions to lessen the physical and psychological stress that such pandemics elicit.

## Figures and Tables

**Table 1:** General characteristics of the study participants.

Parameter	Total (*n* = 254)
**Age, years**	31.7 ± 8.8
**Females**	199 (78.4)
**Marital status**	
Single	121 (47.6)
Married	123 (48.4)
Separated/divorced	10 (3.9)
**Lockdown > 40 days**	201 (79.1)
**Living region**	
Western Region	171 (67.3)
Central Region	49 (19.3)
Eastern Region	22 (8.7)
Others	12 (4.7)
**Education** (BSc. Degree or higher)	231 (91.0)
**Employment status**	
Government	77 (30.3)
Private sector	40 (15.8)
Student	65 (25.6)
Unemployed	35 (13.8)
Other	37 (14.6)
**Has children**	114 (44.9)
**Nationality**	
Saudi	224 (88.2)
**Current smokers**	34 (13.4)
**BMI, kg/m^2^**	26.2 ± 6.5
Underweight, BMI < 18.5 Healthy weight, 18.5 ≤ BMI < 25 Overweight obesity, BMI ≥ 25	20 (7.9) 109 (42.9) 125 (49.2)
**Weight change during confinement**	
Increased	75 (29.5)
Decreased	78 (30.7)
Same/do not know	101 (39.8)

Data are presented as n (%) except for age and BMI as mean ± SD.

**Table 2:** Stress and lifestyle factors before and during home confinement.

Parameter	Before	During	*p*-Value
**Stress score**	10.7 ± 7.3	12.8 ± 7.5	<0.0001
**PSQI score**	6.29 ± 3.27	7.28 ± 3.50	<0.0001
**Physical activity**			
Days of vigorous activity	0 (0, 2)	0 (0, 2)	0.703
Days of moderate activity	0 (0, 2)	0 (0, 2)	0.506
Days of walking for at least 10 min	2 (0, 4)	1 (0, 3)	<0.001
**Screen time**			
Never	4 (1.6)	1 (0.4)	<0.001
1–3 h	84 (33.1)	28 (11.0)	
4–6 h	105 (41.3)	77 (30.3)	
>6 h	61 (24.0)	148 (58.3)	
**Number of snacks**			
<2 snacks	132 (52.0)	116 (45.7)	0.048
≥2 snacks	122 (48.0)	138 (54.3)	
**Eating at the same time**			<0.001
Always/most of the time	173 (68.1)	139 (54.7)	
Sometimes	61 (24.0)	81 (31.9)	
Never/almost never	20 (7.9)	34 (13.4)	
**Supplement use**			
Yes	139 (54.7)	144 (56.7)	0.424
No	115 (45.3)	110 (43.3)	
**Main meals**			0.012
1–2 meals	128 (50.4)	152 (59.8)	
≥3 meals	126 (49.6)	102 (40.2)	
**Eating outside foods**			<0.001
Never/less than once a week	95 (37.4)	217 (85.4)	
1–2 per week	111 (43.7)	26 (10.2)	
≥3 per week	48 (18.9)	11 (4.3)	

Data are presented as mean ± SD (for stress) and median (25, 75th percentile) for physical activity. McNemar’s test was used to compare proportions. Paired *t*-test was used to compares stress and PSQI scores before and during confinement, while Wilcoxon signed-rank test was used for physical activity.

**Table 3:** Linear regression of stress scores during home confinement with participants characteristics and lifestyle factors.

Parameter	Bivariate B (95% CI)	Multiple B (95% CI)
**Age, y** **ear**	−0.096 (−0.20, 0.01)	−0.03 (−0.12, 0.05)
**Sex**		---
Female	0	
Male	−1.24 (−3.51, 1.02)	
**Married (yes vs. no)**	−1.04 (−2.91, 0.82)	---
**Has children (yes vs. no)**	−1.97 (−3.83, −0.10) *	0.22 (−1.30, 1.74)
**Current smoker (yes vs. no)**	0.31 (−2.43, 3.06)	---
**Region**		
Western region	0	0
Central region	−0.49 (−2.88, 1.91)	−0.28 (−1.86, 1.31)
Eastern region	−0.57 (−3.92, 2.78)	−0.37 (−2.55, 1.81)
Others	5.00 (0.58, 9.41) *	−0.14 (−3.05, 2.78)
**Education (BSc. or higher)**	0.64 (−2.61, 3.90)	---
**Employment status**		---
Government	0	
Private	−0.36 (−3.28, 2.55)	
Student	−0.02 (−2.54, 2.50)	
Others	−0.55 (−3.00, 1.90)	
**BMI**	0.00 (−0.14, 0.15)	---
**Weight change (yes vs. no)**	1.93 (0.04, 3.82) *	0.91 (−0.37, 2.19)
**Stress scores before confinements**	0.74 (0.65, 0.83) **	0.67 (0.58, 0.76) **
**PSQI during confinement**	0.93 (0.69, 1.17) **	0.53 (0.34, 0.71) **
**Days of walking for at least 10 min**	−0.44 (−0.84, −0.03) *	−0.42 (−0.69, −0.15) **

* *p* < 0.05; ** *p* < 0.01.

**Table 4:** Association of stress scores with eating habits during home confinement.

Outcome	Model 1 OR (95% CI)	*p*	Model 2 OR (95% CI)	*p*
**(A)** **Eating at the same time**				
Always/most of the time	1 (reference group)		1 (reference group)	
Sometimes	1.07 (1.02, 1.11)	0.002	1.07 (1.02, 1.12)	0.005
Never/almost never	1.11 (1.05, 1.18)	<0.001	1.09 (1.02, 1.16)	0.008
**(B)** **Number of main meals**				
1–2 meals	1 (reference group)		1 (reference group)	
≥3 meals	0.94 (0.91, 0.98)	0.002	0.95 (0.91, 0.99)	0.013
**(C)** **Eating outside food**				
Never/less than once a week	1 (reference group)		1 (reference group)	
1–2 times per week	0.96 (0.90, 1.02)	0.201	0.95 (0.89, 1.02)	0.148
≥3 times per week	1.12 (1.03, 1.22)	0.009	1.08 (0.99, 1.19)	0.076

Logistic regression (n = 254) was used for all outcomes: A, B, and C. Model 1 is adjusted for dietary habits before home confinement. Model 2 is adjusted for (A) smoking, sleep quality, fasting during Ramadan, and eating time before home confinement; (B) age, smoking, sleep quality during confinement, currently fasting during Ramadan, and the number of meals before confinement; (C) sex, BMI, marital status, sleep quality during confinement, currently fasting during Ramadan, smoking, and eating outside before confinement. An OR of 1.11 indicates that the odds of never/almost never eating at the same time during home confinement are 1.11 times higher with 1-unit increase in stress scores after holding smoking, sleep quality, fasting during Ramadan, and eating time before home confinement at fixed values.

**Table 5:** Predictors of weight gain or loss during home confinement.

Variables	OR (95% CI)	*p*
Weight Gain (reference: no weight gain)		
BMI, kg/m^2^	1.03 (0.98, 1.08)	0.211
Sex (male vs. females)	1.54 (0.77, 3.08)	0.224
Married (yes vs. no)	2.40 (1.31, 4.42)	0.005
PSQI during confinement	1.10 (1.01, 1.20)	0.025
Days of walking for at least 10 min	0.82 (0.70, 0.95)	0.007
Number of snacks (≥2 snacks vs. <2 snacks)	1.83 (0.98, 3.42)	0.056
Number of main meals (≥3 meals vs. 1–2 meals)	1.76 (0.95, 3.26)	0.07
Weight loss (Reference: no weight loss)		
Age, years	0.97 (0.93, 1.02)	0.228
Married (yes vs. no)	0.93 (0.46, 1.87)	0.833
Number of main meals (≥3 meals vs. 1–2 meals)	0.36 (0.19, 0.67)	0.001
Current smoker (yes vs. no)	0.49 (0.19, 1.29)	0.149
Days of moderate Activity	1.25 (1.07, 1.46)	0.004

Multinomial logistic regression (n = 254) was used.

## Data Availability

Data can be provided upon request from the corresponding author.

## References

[1] Director-General W. WHO Director-General’s Opening Remarks at the Media Briefing on COVID-19-11 March 2020. https://www.who.int/director-general/speeches/detail/who-director-general-s-opening-remarks-at-the-media-briefing-on-covid-19---11-march-2020.

[2] Khan A., Alsofayan Y., Alahmari A., Alowais J., Algwizani A., Alserehi H., Assiri A., Jokhdar H. (2021). COVID-19 in Saudi Arabia: The National Health Response. East. Mediterr. Health J..

[3] Coronavirus Disease 2019 (COVID-19) Situation Report–51. https://www.who.int/publications/m/item/situation-report---51.

[4] Alhusseini N., Alammari D., Ramadan M., Ziadeh N., Zyadeh Z., Alshamrani J., Qasim H., Alamri N., Alqahtani S.A. (2022). The Impact of COVID-19 Pandemic on Lifestyle among the Saudi Population. J. Public Health Res..

[5] Pellegrini M., Ponzo V., Rosato R., Scumaci E., Goitre I., Benso A., Belcastro S., Crespi C., Michieli F.D., Ghigo E. (2020). Changes in Weight and Nutritional Habits in Adults with Obesity during the “Lockdown” Period Caused by the COVID-19 Virus Emergency. Nutrients.

[6] Cheikh Ismail L., Osaili T.M., Mohamad M.N., Al Marzouqi A., Jarrar A.H., Zampelas A., Habib-Mourad C., Omar Abu Jamous D., Ali H.I., Al Sabbah H. (2021). Assessment of Eating Habits and Lifestyle during the Coronavirus 2019 Pandemic in the Middle East and North Africa Region: A Cross-Sectional Study. Br. J. Nutr..

[7] Alghamdi B.S., Alatawi Y., Alshehri F.S., Tayeb H.O., Tarazi F.I. (2021). Relationship Between Public Mental Health and Immune Status During the COVID-19 Pandemic: Cross-Sectional Data from Saudi Arabia. Risk Manag. Healthc. Policy.

[8] López-Moreno M., López M.T.I., Miguel M., Garcés-Rimón M. (2020). Physical and Psychological Effects Related to Food Habits and Lifestyle Changes Derived from Covid-19 Home Confinement in the Spanish Population. Nutrients.

[9] Brooks S.K., Webster R.K., Smith L.E., Woodland L., Wessely S., Greenberg N., Rubin G.J. (2020). The Psychological Impact of Quarantine and How to Reduce It: Rapid Review of the Evidence. Lancet.

[10] Liu Y.Z., Wang Y.X., Jiang C.L. (2017). Inflammation: The Common Pathway of Stress-Related Diseases. Front. Hum. Neurosci..

[11] Bacaro V., Ballesio A., Cerolini S., Vacca M., Poggiogalle E., Donini L.M., Lucidi F., Lombardo C. (2020). Sleep Duration and Obesity in Adulthood: An Updated Systematic Review and Meta-Analysis. Obes. Res. Clin. Pract..

[12] Block J.P., He Y., Zaslavsky A.M., Ding L., Ayanian J.Z. (2009). Psychosocial Stress and Change in Weight among US Adults. Am. J. Epidemiol..

[13] Ismail L.C., Osaili T.M., Mohamad M.N., Marzouqi A.A., Jarrar A.H., Jamous D.O.A., Magriplis E., Ali H.I., Sabbah H.A., Hasan H. (2020). Eating Habits and Lifestyle during COVID-19 Lockdown in the United Arab Emirates: A Cross-Sectional Study. Nutrients.

[14] Giacalone D., Frøst M.B., Rodríguez-Pérez C. (2020). Reported Changes in Dietary Habits During the COVID-19 Lockdown in the Danish Population: The Danish COVIDiet Study. Front. Nutr..

[15] Madan J., Blonquist T., Rao E., Marwaha A., Mehra J., Bharti R., Sharma N., Samaddar R., Pandey S., Mah E. (2021). Effect of COVID-19 Pandemic-Induced Dietary and Lifestyle Changes and Their Associations with Perceived Health Status and Self-Reported Body Weight Changes in India: A Cross-Sectional Survey. Nutrients.

[16] Al-Musharaf S., Aljuraiban G., Bogis R., Alnafisah R., Aldhwayan M., Tahrani A. (2021). Lifestyle Changes Associated with COVID-19 Quarantine among Young Saudi Women: A Prospective Study. PLoS ONE.

[17] Flanagan E.W., Beyl R.A., Fearnbach S.N., Altazan A.D., Martin C.K., Redman L.M. (2021). The Impact of COVID-19 Stay-At-Home Orders on Health Behaviors in Adults. Obesity.

[18] Khubchandani J., Kandiah J., Saiki D. (2020). The COVID-19 Pandemic, Stress, and Eating Practices in the United States. Eur. J. Investig. Health Psychol. Educ..

[19] Franceschini C., Musetti A., Zenesini C., Palagini L., Scarpelli S., Quattropani M.C., Lenzo V., Freda M.F., Lemmo D., Vegni E. (2020). Poor Sleep Quality and Its Consequences on Mental Health during the COVID-19 Lockdown in Italy. Front. Psychol..

[20] Agurto H.S., Alcantara-Diaz A.L., Espinet-Coll E., Toro-Huamanchumo C.J. (2021). Eating Habits, Lifestyle Behaviors and Stress during the COVID-19 Pandemic Quarantine among Peruvian Adults. PeerJ.

[21] Zachary Z., Brianna F., Brianna L., Garrett P., Jade W., Alyssa D., Mikayla K. (2020). Self-Quarantine and Weight Gain Related Risk Factors during the COVID-19 Pandemic. Obes. Res. Clin. Pract..

[22] Khan A., Alahmari A., Almuzaini Y., Alturki N., Aburas A., Alamri F.A., Albagami M., Alzaid M., Alharbi T., Alomar R. (2021). The Role of Digital Technology in Responding to COVID-19 Pandemic: Saudi Arabia’s Experience. Risk Manag. Heal. Policy.

[23] Sedov I.D., Cameron E.E., Madigan S., Tomfohr-Madsen L.M. (2018). Sleep Quality during Pregnancy: A Meta-Analysis. Sleep Med. Rev..

[24] Demirci J.R., Braxter B.J., Chasens E.R. (2012). Breastfeeding and Short Sleep Duration in Mothers and 6–11-Month-Old Infants. Infant. Behav. Dev..

[25] Guardino C.M., Dunkel Schetter C. (2014). Coping during Pregnancy: A Systematic Review and Recommendations. Health Psychol. Rev..

[26] IPAQ Group Guidelines for the Data Processing and Analysis of the International Physical Activity Questionnaire. https://sites.google.com/view/ipaq/score.

[27] World Health Organization STEPS Manual: Section 5: Collecting Step 2 Data: Physical Measurements. https://cdn.who.int/media/docs/default-source/ncds/ncd-surveillance/steps/part3-section5.pdf?sfvrsn=a46653c7_2.

[28] Buysse D.J., Reynolds C.F., Charles F., Monk T.H., Berman S.R., Kupfer D.J. (1989). Pittsburgh Sleep Quality Index (PSQI). Psychiatry Res..

[29] Carlson L.E., Cherian Thomas B. (2007). Development of the Calgary Symptoms of Stress Inventory (C-SOSI). Int. J. Behav. Med. Copyr. C.

[30] Peduzzi P., Concato J., Kemper E., Holford T.R., Feinstein A.R. (1996). A Simulation Study of the Number of Events per Variable in Logistic Regression Analysis. J. Clin. Epidemiol..

[31] Alfawaz H., Amer O.E., Aljumah A.A., Aldisi D.A., Enani M.A., Aljohani N.J., Alotaibi N.H., Alshingetti N., Alomar S.Y., Khattak M.N.K. (2021). Effects of Home Quarantine during COVID-19 Lockdown on Physical Activity and Dietary Habits of Adults in Saudi Arabia. Sci. Rep..

[32] Cellini N., Canale N., Mioni G., Costa S. (2020). Changes in Sleep Pattern, Sense of Time and Digital Media Use during COVID-19 Lockdown in Italy. J. Sleep Res..

[33] Alqahtani S.S., Banji D., Banji O.J.F.F. (2021). A Survey Assessing Sleep Efficiency among Saudis during COVID-19 Home Confinement Using the Pittsburgh Sleep Quality Index: A Call for Health Education. Saudi. Pharm. J..

[34] Ammar A., Trabelsi K., Brach M., Chtourou H., Boukhris O., Masmoudi L., Bouaziz B., Bentlage E., How D., Ahmed M. (2020). Effects of Home Confinement on Mental Health and Lifestyle Behaviours during the COVID-19 Outbreak: Insight from the ECLB-COVID19 Multicenter Study. Biol. Sport.

[35] Alenazi T.H., BinDhim N.F., Alenazi M.H., Tamim H., Almagrabi R.S., Aljohani S.M., Basyouni M.H., Almubark R.A., Althumiri N.A., Alqahtani S.A. (2020). Prevalence and Predictors of Anxiety among Healthcare Workers in Saudi Arabia during the COVID-19 Pandemic. J. Infect. Public Health.

[36] Alhusseini N., Alqahtani A. (2020). COVID-19 Pandemic’s Impact on Eating Habits in Saudi Arabia. J. Public Health Res..

[37] Tomiyama A.J. (2019). Stress and Obesity. Annu. Rev. Psychol.

[38] Costarelli V., Michou M. (2023). Perceived Stress Negatively Affects Diet Quality and Life Satisfaction during the COVID-19 Lockdown Period, in Greece. Nutr. Food Sci..

[39] Chaput J.P., McHill A.W., Cox R.C., Broussard J.L., Dutil C., da Costa B.G.G., Sampasa-Kanyinga H., Wright K.P. (2022). The Role of Insufficient Sleep and Circadian Misalignment in Obesity. Nat. Rev. Endocrinol..

[40] BaHammam A.S., Pirzada A. (2023). Timing Matters: The Interplay between Early Mealtime, Circadian Rhythms, Gene Expression, Circadian Hormones, and Metabolism—A Narrative Review. Clocks Sleep.

[41] Binks H., Vincent G.E., Gupta C., Irwin C., Khalesi S. (2020). Effects of Diet on Sleep: A Narrative Review. Nutrients.

[42] Schroor M.M., Sennels H.P., Fahrenkrug J., Jørgensen H.L., Plat J., Mensink R.P. (2019). Diurnal Variation of Markers for Cholesterol Synthesis, Cholesterol Absorption, and Bile Acid Synthesis: A Systematic Review and the Bispebjerg Study of Diurnal Variations. Nutrients.

[43] Dashti H.S., Scheer F.A.J.L., Saxena R., Garaulet M. (2019). Timing of Food Intake: Identifying Contributing Factors to Design Effective Interventions. Adv. Nutr..

[44] Renzo L.D., Gualtieri P., Cinelli G., Bigioni G., Soldati L., Attinà A., Bianco F.F., Caparello G., Camodeca V., Carrano E. (2020). Psychological Aspects and Eating Habits during COVID-19 Home Confinement: Results of EHLC-COVID-19 Italian Online Survey. Nutrients.

[45] Verma S., Mishra A. (2020). Depression, Anxiety, and Stress and Socio-Demographic Correlates among General Indian Public during COVID-19. Int. J. Soc. Psychiatry.

[46] Abril-Ulloa V., Santos S.P.L.d., Morejón-Terán Y.A., Carpio-Arias T.V., Espinoza-Fajardo A.C., Vinueza-Veloz M.F. (2022). Stress and Diet Quality Among Ecuadorian Adults During the COVID-19 Pandemic. A Cross-Sectional Study. Front. Nutr..

[47] Scarmozzino F., Visioli F. (2020). COVID-19 and the Subsequent Lockdown Modified Dietary Habits of Almost Half the Population in an Italian Sample. Foods.

[48] Tayyem R., Ibrahim M.O., Mortada H., Alkhalaf M., Bookari K., Al Sabbah H., Qasrawi R., Kamel I., Dashti S., Allehdan S. (2022). Sex Disparities in Food Consumption Patterns, Dietary Diversity and Determinants of Self-Reported Body Weight Changes before and amid the COVID-19 Pandemic in 10 Arab Countries. Front. Public Health.

[49] Ferrante G., Camussi E., Piccinelli C., Senore C., Armaroli P., Ortale A., Garena F., Giordano L. (2020). Did Social Isolation during the SARS-CoV-2 Epidemic Have an Impact on the Lifestyles of Citizens?. Epidemiol. Prev..

[50] Soltani H., Keim N.L., Laugero K.D. (2018). Diet Quality for Sodium and Vegetables Mediate Effects of Whole Food Diets on 8-Week Changes in Stress Load. Nutrients.

[51] Soltani H., Keim N.L., Laugero K.D. (2019). Increasing Dietary Carbohydrate as Part of a Healthy Whole Food Diet Intervention Dampens Eight Week Changes in Salivary Cortisol and Cortisol Responsiveness. Nutrients.

[52] DeNicola E., Aburizaiza O.S., Siddique A., Khwaja H., Carpenter D.O. (2015). Obesity and Public Health in the Kingdom of Saudi Arabia. Rev Env. Health.

[53] World Health Survey-Saudi Arabia. https://www.moh.gov.sa/en/Ministry/Statistics/Population-Health-Indicators/Documents/World-Health-Survey-Saudi-Arabia.pdf.

[54] MOI Agency for Security Capabilities All Precautionary Measures Continued till the End of Ramadan, a Complete Curfew During the Period during 30/09-4/10/1441 Ah. https://www.moi.gov.sa/wps/portal/Home/sectors/mafpasd/contents/!ut/p/z1/jZHNTsMwEIRfJZccK49_QszRAhG7REItCg2-RFaJWiPi0BI18PYkoVfS-rDyjr_ZXa2JJSWxwZ38znW-De5jyF_tTUXpvdQsQy6FZFCp4Utj1jAyIZsJuMuUFmkOyDxLYJQu1rcrzqE4sdf48c9RuM4_A9j58s_uSJaXmgxb8O-Hg1XEbtvQ1d.

[55] Saudi Arabia Announces 24-Hour Curfew for Eid Al-Fitr Holiday. Arab News. https://www.arabnews.com/node/1673736/saudi-arabia.

[56] Reuters Staff Saudi Arabia to Introduce 24-Hour Daily Curfew across the Kingdom for Eid Holiday. Reuters. https://www.reuters.com/article/health-coronavirus-saudi-curfew/saudi-arabia-to-introduce-24-hour-daily-curfew-across-the-kingdom-for-eid-holiday-idUSL8N2CU7AC.

[57] Almeneessier A.S., Bahammam A.S. (2018). How Does Diurnal Intermittent Fasting Impact Sleep, Daytime Sleepiness, and Markers of the Biological Clock? Current Insights. Nat. Sci. Sleep.

[58] Osman F., Haldar S., Henry C.J. (2020). Effects of Time-Restricted Feeding during Ramadan on Dietary Intake, Body Composition and Metabolic Outcomes. Nutrients.

[59] Akbari H.A., Yoosefi M., Pourabbas M., Weiss K., Knechtle B., Vancini R.L., Trakada G., Saad H.B., Lavie C.J., Ghram A. (2022). Association of Ramadan Participation with Psychological Parameters: A Cross-Sectional Study during the COVID-19 Pandemic in Iran. J. Clin. Med..

[60] Renzo L.D., Gualtieri P., Pivari F., Soldati L., Attinà A., Cinelli G., Esposito E., Leggeri C., Caparello G., Barrea L. (2020). Eating Habits and Lifestyle Changes during COVID-19 Lockdown: An Italian Survey. J. Transl. Med..

[61] Baquerizo-Sedano L., Chaquila J.A., Aguilar L., Ordovás J.M., González-Muniesa P., Garaulet M. (2021). Anti-COVID-19 Measures Threaten Our Healthy Body Weight: Changes in Sleep and External Synchronizers of Circadian Clocks during Confinement. Clin. Nutr..

[62] Alshahrani S.M., Alghannam A.F., Taha N., Alqahtani S.S., Al-Mutairi A., Al-Saud N., Alghnam S. (2022). The Impact of COVID-19 Pandemic on Weight and Body Mass Index in Saudi Arabia: A Longitudinal Study. Front. Public Health.

[63] Mason T.B., Barrington-Trimis J., Leventhal A.M. (2021). Eating to Cope With the COVID-19 Pandemic and Body Weight Change in Young Adults. J. Adolesc. Health.

[64] The World Bank Group Individuals Using the Internet (% of Population)-Saudi Arabia. Online Resource, by The World Bank. https://data.worldbank.org/indicator/IT.NET.USER.ZS?end=2019&locations=SA&start=200%200.

